# Extensive Coronary Thrombosis as a Sequelae of COVID-19 Presenting as a STEMI

**DOI:** 10.7759/cureus.15258

**Published:** 2021-05-26

**Authors:** Muhammad Madkour, Javad Savoj, Syed Iftikhar, Alain Waked

**Affiliations:** 1 Internal Medicine, University of California Riverside School of Medicine/Riverside Community Hospital, Riverside, USA; 2 Cardiology, University of California Riverside School of Medicine/Riverside Community Hospital, Riverside, USA; 3 Interventional Cardiology, Riverside Community Hospital, Riverside, USA

**Keywords:** covid19, coronary artery thrombosis, st-segment elevation myocardial infarction (stemi), coronary artery angiography, hypercoagulable

## Abstract

Thrombotic complications, in particular ST-elevation myocardial infarction (STEMI), have been described in active Coronavirus disease 2019 (COVID-19) cases. This is a result of systemic inflammation that often leads to endothelial activation, microvascular thrombosis and a hypercoagulable state. However, it is unknown how long patients who have cleared the COVID-19 infection remain at risk for coronary thrombosis as a sequela of disease burden. We present a case of a patient who presented three to four weeks following the initial infection.

## Introduction

Severe acute respiratory syndrome coronavirus 2 (SARS-CoV-2) virus predominantly causes respiratory illness; however, cardiovascular complications, such as myocardial injury, arrhythmias, acute heart failure and venous thromboembolism are occurring more frequently than previously suspected. The pathophysiology of cardiovascular injury in patients with Coronavirus disease 2019 (COVID-19) is hypothesized to occur through a combination of direct invasion of the myocardial tissue, cytokine storm, and a prothrombotic state leading to microthrombi formation [1-3]. Given the multitude of cardiovascular complications, myocardial infarctions in particular, ST-elevation myocardial infarction (STEMI) is poorly understood in COVID-19 [2]. Of the STEMI cases that have been reported with active COVID-19 infection, there is an increased incidence of acute thrombosis noted during initial catheterization, which is often attributed to increased inflammation, microvascular coagulopathy, and platelet aggregation [1,2]. However, in patients who have successfully cleared the infection, we find that they can still be at risk for prothrombotic processes leading to a STEMI.

## Case presentation

A 43-year-old Vietnamese-American man with no known medical history, having multiple COVID-19-positive family contacts at home, presented with chest pain for two days. Approximately three to four weeks prior to presentation, he experienced fever, cough, and extreme body aches. The patient had previously been in good health and takes no medications. He denies tobacco/alcohol use and any family history of early cardiac disease or cardiac-related deaths.

**Figure 1:**
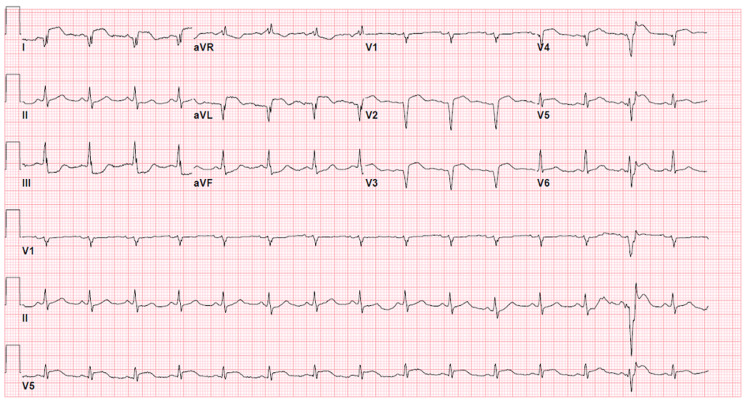
12-lead electrocardiogram shows 4 mm ST elevations in leads I and aVL with reciprocal ST depressions inferiorly, which is compatible with an inferior-lateral ST-segment elevation myocardial infarction.

On initial assessment, the patient was in moderate acute distress. His vital signs were stable, and oxygen saturation was >94% on room air. A cardiac exam revealed regular heart sounds with no murmur. A pulmonary exam revealed bilateral diffuse rhonchi. No jugular venous distension or lower extremity edema was noted. Peak troponin I was >200 ng/mL (normal <0.08 ng/mL). Other laboratory values were significant for elevated C-reactive protein of 21.4 mg/dL, ferritin of 1993 ng/mL, D-dimer of 2037 ng/mL, and lactate dehydrogenase of 1382 U/L. An initial SARS-CoV-19 polymerase chain reaction (PCR) was negative. Lipid profile, including total cholesterol, low-density lipoprotein (LDL) and triglyceride levels were within normal limits. Hemoglobin A1c was 5.2%. Chest X-ray showed bilateral, patchy, peripherally dominant airspace opacities consistent with multifocal pneumonia. An electrocardiogram showed ST segment elevations in leads I and aVL, with reciprocal ST depressions inferiorly, and nonspecific ST elevations with Q waves in leads V1-V4 (Figure 1). Code STEMI was activated.

**Figure 2:**
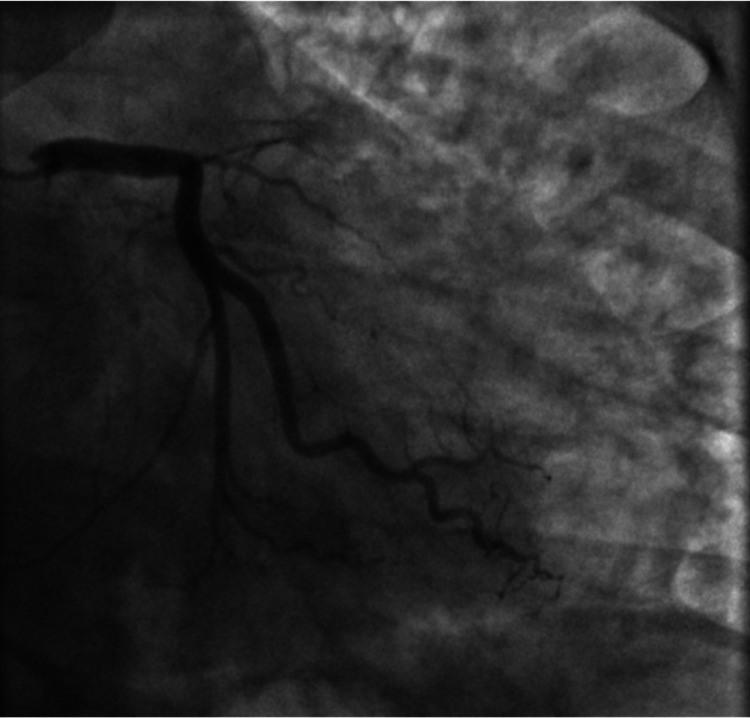
Right anterior oblique (RAO) coronary angiographic image showing complete 100% ostial occlusion of the left anterior descending (LAD).

**Figure 3:**
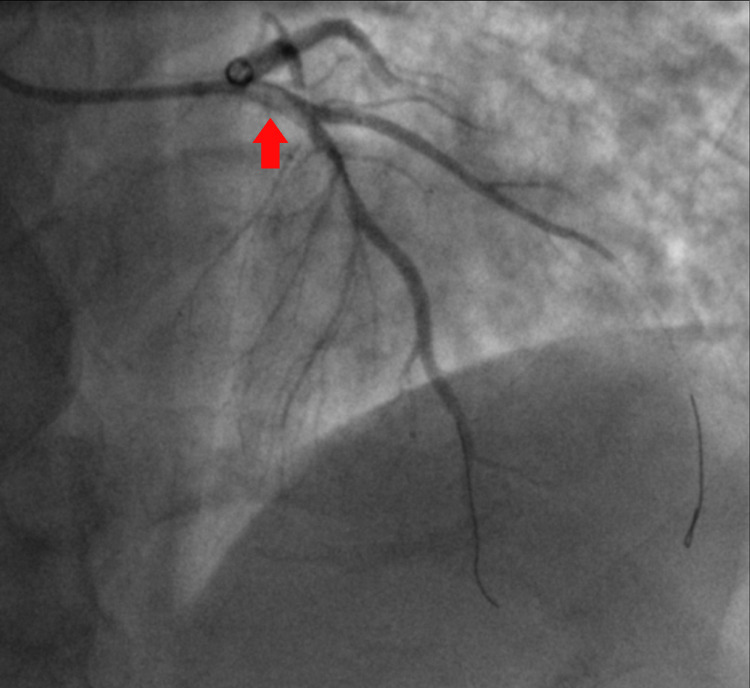
Left anterior oblique (LAO) cranial coronary angiographic image showing extensive coronary thrombosis noted at the proximal and distal left anterior descending (LAD) impeding flow.

**Figure 4:**
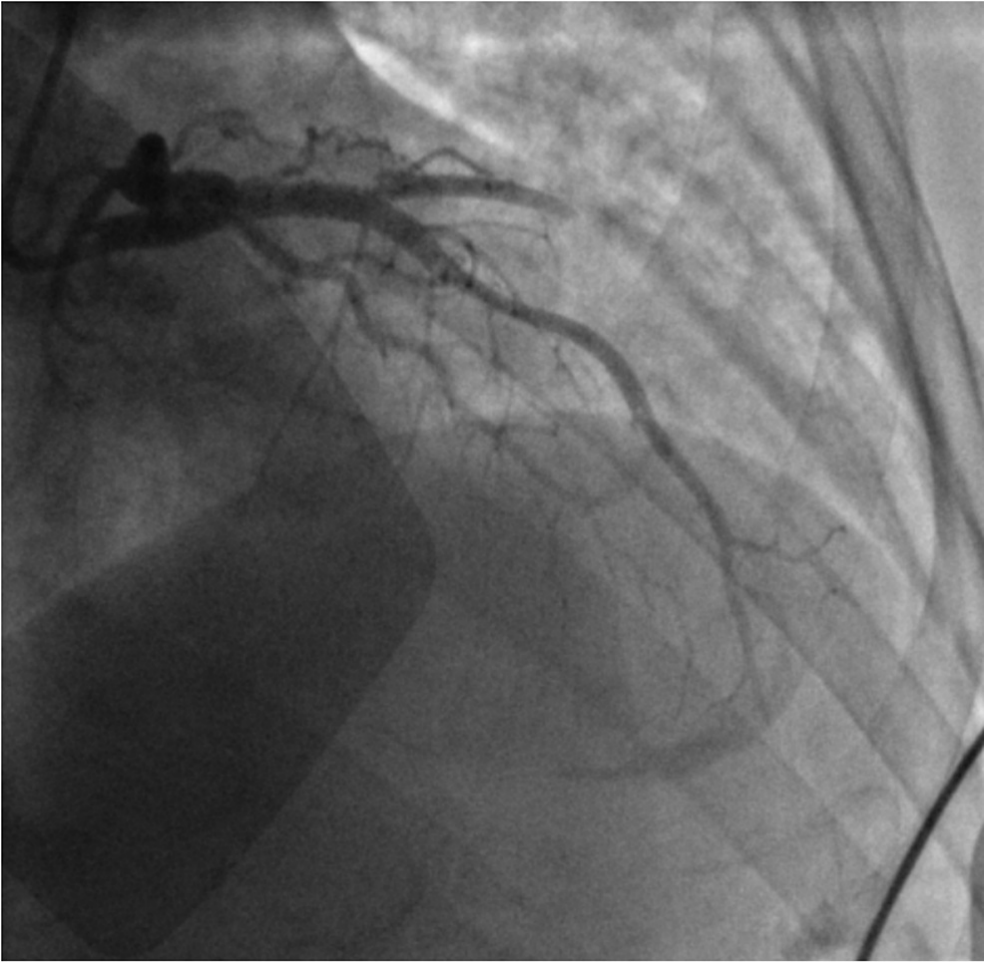
Right anterior oblique (RAO) cranial coronary angiographic image post aspiration thrombectomy and post percutaneous cutaneous intervention (PCI) stent to the proximal left anterior descending (LAD).

Emergent cardiac catheterization showed 100% ostial thrombotic occlusion of the left anterior descending (LAD) coronary artery (Figure 2). Extensive coronary thrombosis impeding flow was noted at the proximal and distal LAD (Figure 3). As a result, multiple rounds of aspiration thrombectomy were performed, intracoronary glycoprotein IIb/IIIa inhibitors were administered, and a drug-eluting stent was placed in the proximal LAD, resulting in thrombolysis in myocardial ischemia (TIMI) III flow (Figure 4). Left ventricular end-diastolic pressure was measured to be 28-33 mmHg. An intra-aortic balloon pump was placed for circulatory support, and the patient was monitored in the intensive care unit. Initial transthoracic echocardiogram (TTE) showed akinesis in LAD distribution of the anterior apical wall and estimated ejection fraction of 25%, with no significant valvular pathology.

Repeat TTE during the same hospitalization revealed an ejection fraction of 30%-35%, and a wearable cardioverter defibrillator was recommended for primary prevention. The patient was retested for COVID-19, and the SARS-CoV-19 PCR result was negative twice more. COVID-19 antibody was found to be positive.

Following hospital discharge, the patient was followed closely in clinic. He reported return to baseline activity and was now able to jog on his treadmill symptom-free. Outpatient TTE at 45-day follow-up showed significant improvement in left ventricular ejection fraction, measured at 45%-50%. He continues to remain on dual antiplatelet therapy with aspirin and ticagrelor for the LAD stent as per guidelines.

## Discussion

Patients with COVID-19 who require hospitalization typically present with significant respiratory symptoms. However, as more patients have succumbed to the virus, we are observing a wide range of cardiovascular manifestations that could concomitantly occur. Whereas myocardial injury from myocarditis or hemodynamic alterations can be common, the incidence of plaque rupture acute coronary syndrome in COVID-19, particularly after a cleared infection, remains unclear. Many mechanisms contribute to high-risk plaque destabilization and link systemic viral infection with acute coronary ischemic syndromes [4].

After an exhaustive literature search for case reports of patients who subsequently developed STEMI after a cleared COVID-19 infection, we found that this area was limited. In one retrospective analysis from Italy in June 2020, data were collected from all confirmed patients with active COVID-19 who underwent coronary angiography due to STEMI. It was found that a STEMI might represent the first clinical manifestation of active ongoing infection; however, in approximately 40% of the cases, a culprit lesion was not identifiable by angiography [5]. The literature was scarce in describing patients similar to the one we presented here.

Active COVID-19 infection is known to lead to systemic inflammation that could in turn result in dysregulation of coronary vascular endothelial function and ultimately cause vasoconstriction and thrombosis [1,2,6]. However, as observed in our patient with no underlying cardiovascular risk factors (lipid profile and hemoglobin A1c were within normal limits), his prior symptoms and laboratory confirmation tests were consistent with a cleared COVID-19 infection. Even after clearing the infection, the patient remained at risk for coronary thrombosis as a result of the known hypercoagulable state associated with the disease process, and not the virus itself. It is unknown how long patients with cleared COVID-19 remain at risk for coronary thrombosis. This lingering impact of endothelial activation and hypercoagulability leading to myocardial injury supports the need for ongoing investigation and longitudinal follow-up to evaluate the long-term cardiac consequences of COVID-19 infection.

## Conclusions

COVID-19 has been associated with increased cardiovascular injury. Although the exact pathophysiology of cardiac injury remains unclear, it is important to promptly recognize these manifestations. A high index of suspicion must be maintained for all patients who have tested positive for COVID-19 and who develop new-onset chest pain, given these patients remain at risk for coronary thrombosis from the known hypercoagulable state. Due to the potentially life-threatening nature of a STEMI, rapid diagnosis and treatment are paramount. Our case of a patient with cleared COVID-19 infection and no known cardiovascular risk factors who presented with coronary thrombosis illustrates the need for further longitudinal follow-up, given COVID-19 continues to cause significant morbidity and mortality globally.
